# Treating adolescent pseudomyopia and elevated intraocular pressure using chiropractic and moxibustion: A CARE-compliant case report

**DOI:** 10.1097/MD.0000000000037564

**Published:** 2024-03-15

**Authors:** Yiyan Fang, Zengtu Li, Hantong Hu, Ziyu Ye

**Affiliations:** a Zhejiang Chinese Medical University, Hangzhou City, China; b Department of Acupuncture and Moxibustion, Pujiang County Traditional Chinese Medicine Hospital, Jinhua City, China; c Department of Tuina, the Third Affiliated Hospital of Zhejiang Chinese Medical University, Hangzhou City, China; d Department of Acupuncture and Moxibustion, the Third Affiliated Hospital of Zhejiang Chinese Medical University, Hangzhou City, China; e Clinical Medical College of Acupuncture Moxibustion and Rehabilitation, Guangzhou University of Chinese Medicine, Guangzhou City, China.

**Keywords:** adolescent pseudomyopia, chiropractic, elevated intraocular pressure, moxibustion

## Abstract

**Rationale:**

This case report aims to provide clinical evidence on the effectiveness of integrating chiropractic and moxibustion techniques for treating pseudomyopia accompanied by elevated intraocular pressure resulting from cervical spine issues because the application of complementary medicine modalities for managing such vision disorders currently lacks adequate investigations.

**Patient concerns:**

A 6-year-old patient presented with blurred vision, intermittent ocular discomfort, and upper cervical discomfort.

**Diagnoses:**

Spine-related increased intraocular pressure and pseudomyopia.

**Interventions:**

The patient received integrative treatment of chiropractic and walnut-shell moxibustion 3 times a week for a total of 10 treatment sessions.

**Outcomes:**

The patient exhibited progressive improvements in visual acuity and reductions in intraocular pressure over the treatment period, with unaided vision exceeding 2 lines of improvement in visual acuity charts and normalized intraocular pressure after 10 treatment sessions. These therapeutic effects were sustained at 3-month follow-up.

**Lessons:**

The integrative use of chiropractic and walnut-shell moxibustion demonstrates considerable potential in alleviating symptoms of pseudomyopia, reducing intraocular pressure, and restoring visual function in spine-related pseudomyopia cases.

## 1. Introduction

Myopia is the most prevalent ocular disorder globally and a significant risk factor for various other ocular diseases.^[1,2]^ Myopia management primarily focuses on slowing its progression and preventing its onset. Currently accepted interventions for controlling myopia progression include optical correction, under-correction, contact lens use, and increased outdoor activity exposure.^[3,4]^

A thorough literature review reveals that reports on spine-related pseudomyopia accompanied by elevated intraocular pressure and treated with complementary and alternative medicine are scarce. We present a case of an adolescent patient with pseudomyopia and elevated intraocular pressure, successfully treated through multiple sessions of chiropractic care combined with moxibustion.

## 2. Case presentation

A 6-year-old male patient visited our hospital Department of Massage on May 27, 2020, with a complaint of blurred vision persisting for 1 month. The patient, a frequent reader averaging 6 hours daily, reported no associated symptoms such as eye pain, headache, or dizziness. His medical history was negative for ocular trauma and congenital ocular diseases.

This case study was approved by the Third Affiliated Hospital of Zhejiang Chinese Medical University. As the patient is a minor, written informed consent was obtained from the legal guardian within this specific age group.

### 2.1. Physical examinations at admission

Initial physical examination revealed straightening of the cervical physiological curvature, tenderness in the left paravertebral area of the second cervical vertebra (C2), and rotation in the upper cervical vertebrae. Visual acuity was recorded at 4.6 in the right eye and 4.8 in the left eye. Intraocular pressure measured 22 mm Hg in both eyes, as determined by noncontact tonometry.

### 2.2. Diagnosis

The diagnosis of pseudomyopia with spine-derived elevated intraocular pressure was established based on the patient medical history, physical examination, and eye examination results.

### 2.3. Treatment regimen

The patient underwent a combined treatment of chiropractic therapy and walnut-shell moxibustion for a total of 10 treatment sessions.

#### 2.3.1. The procedures of chiropractic therapy.

The procedure for chiropractic therapy was as follows.

(1) Positioning: The patient lay in a supine position on the treatment bed. The chiropractor sat at the head of the bed.
(2) Initial Assessment: Using the pads of the index, middle, and ring fingers, the chiropractor located the offset point between the spinous process and transverse process. For example, the left offset of the C2 transverse process was identified.
(3) Manipulation Techniques
  • Traction: The chiropractor pressed the left articular process of C2 with the fingertips and gently tilted the patient head to the left, creating static traction for approximately 1 minute.
  • Rotation: The head was then secured with both hands and gently pulled upwards. The chiropractor rotated the head left and right 5 to 10 times.
  • Segmental Movement: Placing the radial edge of the index finger on the posterior occiput and thumbs on either side of the C2 transverse process, both hands worked in coordination to move the upper and lower segments of C2 in various directions.
  • Final Adjustment: With the head rotated maximally to the left and tilted back by 15°, a small-amplitude, rapid pull was applied. The absence of an elastic sound indicated a successful reset.
(4) Duration and Frequency: Each session lasted approximately 5 minutes. The treatment frequency was 3 times a week, completing a course of 10 sessions.

#### 2.3.2. The procedures of walnut-shell moxibustion.

**Figure 1.**
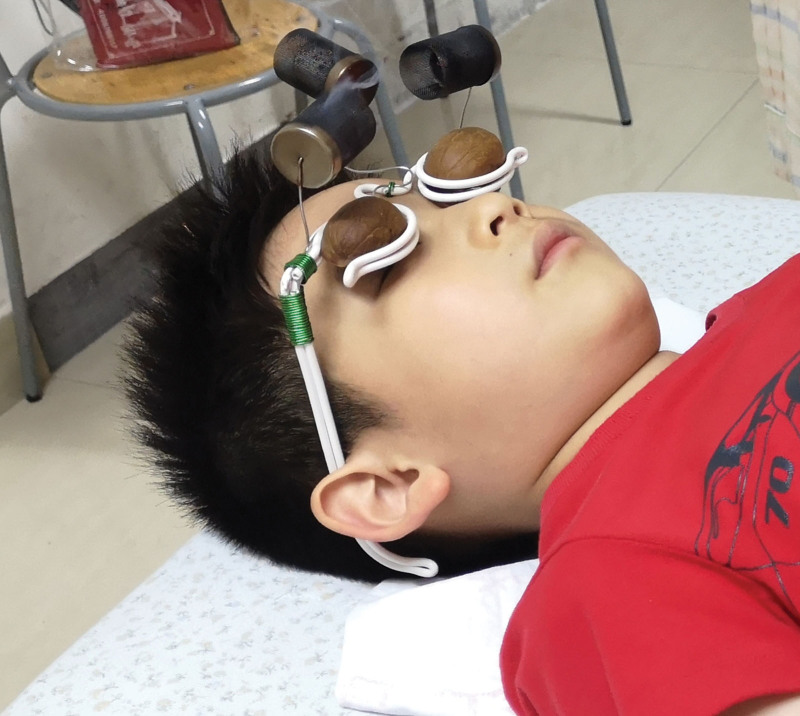
Schematic diagram of walnut-shell moxibustion.

Figure 1 presents a schematic diagram of the walnut-shell moxibustion process. The treatment involved several steps as follows.

1. Preparation: Two semi-circular walnut shells were utilized, each with 5 grams of 2 types of Chinese medicine powder—one for soaking (medicine 1: mint, chrysanthemum, mulberry leaves, etc), and another for filling (medicine 2: Polygonatum sibiricum, Mongolian flower, Lycium barbarum, Scutellaria baicalensis, etc). Additionally, a pair of spectacle frames designed for walnut-shell moxibustion and 2 to 3 segments of a moxa stick were required.
2. Soaking Process: The walnut shells were immersed in boiling water mixed with medicine 1 for 30 minutes.
3. Filling Process: Medicine 2 was converted into a paste thick enough to avoid dripping when inverted. The walnut shells were then filled with this paste to a thickness of approximately 2 mm.
4. Application: The prepared walnut shells were placed onto the spectacle frames, and the moxa stick was lit. The patient lay on their back and wore the spectacle frames.
5. Duration: Each treatment session lasted approximately 20 minutes. The frequency was 3 times a week, with a complete course comprising 10 sessions.

### 2.4. Outcome measures and results

After 3 treatment sessions, the patient visual acuity improved from 4.6 to 4.7 in the right eye and was maintained at 4.8 in the left eye. Intraocular pressure reduced from 22 mm Hg to 18 mm Hg.

After 10 treatment sessions, compared to pretreatment, unaided visual acuity increased from 4.6 to 4.8 (i.e., improved by 2 lines in visual acuity charts) in the right eye and from 4.8 to 5.0 (i.e., improved by 2 lines in visual acuity charts) in the left eye. Intraocular pressure was maintained within the normal range at 18 mm Hg.

At 3-month follow-up, the patient visual acuity and intraocular pressure were sustained at the post-treatment levels.

## 3. Discussion

Myopia, the most common form of ametropia, occurs when the eye, in a relaxed state, focuses parallel light rays in front of the retina, leading to blurred distant vision and clear near vision, often accompanied by visual fatigue.^[5]^ Factors contributing to its development include poor reading and working postures at close range, excessive eye use causing ciliary muscle contraction and spasm, and lens thickening. Clinical manifestations include blurred vision, eye swelling, and dizziness. Untreated, it can progress to true myopia with increased eye axis length.^[6,7]^

Myopia is categorized into pseudomyopia, true myopia, and mixed myopia. For adolescent pseudomyopia, low concentrations of ciliary muscle paralysis agents like atropine are commonly used clinically. However, atropine can lead to complications such as pupil dilation, accommodation paralysis, photophobia, and blurred vision.^[8]^

The patient in this case, presenting with pseudomyopia, elevated intraocular pressure, and neck pain, likely had a cervical spine-derived condition. Currently, such spine-derived vision disorders lack sufficient clinical recognition.

Cervical spine injuries or degenerative changes can disrupt intraocular and extraocular muscle movements, sympathetic nerve function, or cause vasospasm. The pathology links to the posterior occipital muscle group-eyeball movement coupling chain. Cervical spine imbalance leads to visual impairment through this chain. Anterior head positioning to maintain normal vision strains the posterior occipital muscle group, disrupting extraocular muscle movement and causing visual axis imbalance, which strains intraocular muscles, leading to muscle fatigue or ametropia and reduced vision. Additionally, cervical spinal joint dislocation can stimulate or compress the superior cervical sympathetic ganglion and stellate ganglion, affecting vision. Manipulative therapies can relax eye muscles and adjust disordered joints, alleviating pathological couplings and improving blood supply to the eyes, potentially explaining the observed decrease in intraocular pressure and vision improvement.^[9]^

Furthermore, based on traditional Chinese medicine principles, moxibustion is known to warm meridians, dredge collaterals, relax tendons, and enhance blood circulation. In the case presented, moxibustion was safely applied to the eyes using a specialized modality known as “walnut-shell moxibustion.” This technique was combined with a powder formulated to benefit the liver and improve eyesight, comprising ingredients such as mint, chrysanthemum, mulberry leaves, Polygonatum sibiricum, Lycium barbarum, and Scutellaria baicalensis. This combination is believed to clear the meridians and collaterals, as well as the qi and blood surrounding the eyes, thereby alleviating symptoms of eye fatigue and dryness. Moreover, walnut-shell moxibustion not only enhances the potency of the partitioned medicine but also effectively harnesses the synergistic effects of both the moxibustion materials and the medicinal properties of the partition, leading to improved clinical outcomes.^[10]^

However, the limitation of this study should be acknowledged. First, this study is limited by the lack of a control group for comparison. The open-label design is subject to inherent bias. Second, as a single case study, the results have limited generalizability. Third, the follow-up duration is insufficient. Longer-term follow-up would also help establish the durability of treatment effects. Therefore, randomized controlled trials with larger sample sizes and rigorous methodology are required to further verify the efficacy and safety of this treatment modality. Furthermore, the underlying mechanisms need to be investigated through imaging and other diagnostic techniques.

## 4. Conclusions

In conclusion, this patient with spine-related increased intraocular pressure and pseudomyopia was effectively treated by the integrative use of chiropractic and walnut-shell moxibustion. This integrative approach yielded satisfactory outcomes without adverse events, highlighting its potential clinical value. Nonetheless, additional rigorous research with larger sample sizes is required to further verify the efficacy and safety of this treatment modality.

## Acknowledgments

The authors would like to acknowledge the patient who participated in our study.

## Author contributions

**Conceptualization:** Hantong Hu.

**Writing – original draft:** Yiyan Fang, Zengtu Li.

**Writing – review & editing:** Hantong Hu, Ziyu Ye.
